# Supplementation of Underfed Twin-Bearing Ewes with Herbal Vitamins C and E: Impacts on Birth Weight, Postnatal Growth, and Pre-Weaning Survival of the Lambs

**DOI:** 10.3390/ani10040652

**Published:** 2020-04-09

**Authors:** Víctor H. Parraguez, Francisco Sales, Oscar A. Peralta, Eileen Narbona, Raúl Lira, Mónica De los Reyes, Antonio González-Bulnes

**Affiliations:** 1 Faculty of Veterinary Sciences, University of Chile, Santiago 8820808, Chile; operalta@uchile.cl (O.A.P.); eileen.narbona@gmail.com (E.N.); mdlreyes@uchile.cl (M.D.l.R.); 2 Faculty of Agricultural Sciences, University of Chile, Santiago 8820808, Chile; 3 INIA-Kampenaike, Punta Arenas 6212707, Chile; fsales@inia.cl (F.S.); rlira@inia.cl (R.L.); 4 INIA-Madrid, Ciudad Universitaria s/n. 28040 Madrid, Spain; bulnes@inia.es; 5 Facultad de Veterinaria, Universidad Complutense de Madrid, Ciudad Universitaria s/n. 28040 Madrid, Spain

**Keywords:** ovine pregnancy outcome, postnatal growth and survival, antioxidant vitamins

## Abstract

**Simple Summary:**

In twin-bearing ewes, undernourishment during pregnancy lowers the birth weight and increases morbidity and mortality of lambs. One of the causes is oxidative stress, which can be prevented by supplementing antioxidant vitamins. In the present study we tested the effect of supplementation with herbal vitamins C and E alone or in combination with concentrate on pregnancy outcomes and growth and survival of lambs during early postnatal stages (lactation). The results showed that vitamin supplementation increased offspring birth weight and antioxidant capacity, with a trend towards higher body weight (BW) at weaning. Nutritional supplementation only had a positive effect on birth weight, meanwhile combined supplementation of vitamins and concentrate improves the lamb response. Lambs’ survival was not affected by the treatment, although absolute values of survival were higher in vitamin-treated groups. It is concluded that supplementation with herbal vitamins C and E alone or in combination with concentrate during pregnancy may constitute a good nutritional strategy to improve productivity in sheep herds reared in harsh environmental conditions.

**Abstract:**

Twin-bearing pregnancies of sheep reared in harsh environmental conditions result in maternal undernutrition and feto-maternal oxidative stress, leading to intrauterine growth restriction (IUGR). We assessed the efficiency of supplementation with antioxidant herbal vitamins C and E alone or in combination with concentrate throughout gestation on pregnancy outcomes, pre-weaning growth, and survival of twin lambs from grazing ewes at the Magellan Steppe. Four groups (*n* = 30 each) of twin-bearing ewes received a base natural prairie (P) diet, supplemented with either herbal vitamins C 500 mg and E 350 IU per day (V) or concentrated food (S); groups were: P, P + V, P + S, and P + VS. Vitamins and concentrate were supplemented until parturition. At birth, lambs were weighed, and blood was drawn for total antioxidant capacity (TAC) evaluation. Lamb body weight (BW) and survival rate were evaluated at mid-lactation (60 days) and at weaning (120 days). Vitamin supplementation resulted in increased lamb birth weight and TAC, with a trend towards higher BW at weaning, while nutritional supplementation only had a positive effect on birth weight. Lamb survival was higher in both vitamin supplemented groups. In conclusion, supplementation with herbal vitamins C and E alone or in combination with concentrate food during pregnancy may constitute a good nutritional strategy for sheep reared in harsh environmental conditions.

## 1. Introduction

On a global scale, the production of small ruminants is carried out in extensive grazing systems, mainly in arid or semi-arid lands where the contribution of grasslands generally does not cover the nutritional demands of the animals [1]. Based on data from the Food and Agriculture Organization (FAO) [2] and Iñiguez [3], it is estimated that more than 30% of the world’s sheep stock is bred in dry regions, where crops or other livestock production is not possible. Arid and semi-arid regions are characterized by low annual rainfall (<400 mm) and poor water-bearing capacity of the prairies, which lead to low primary productivity [4]. This feed scarcity, mainly during summer and winter, is a major constraint for sheep reared in these regions [5].

The Magellan Patagonia region of Chile constitutes a good example of sheep farming on semi-arid extensive grazing systems. In Chile, around 66% of the national sheep stock are raised in arid and semi-arid regions [6,7], with more than 50% found in the Magellan cold steppe. The Magellan Steppe has low annual precipitation (200–400 mm) and low average temperatures (around 5 °C), high evaporation, and strong winds. All these factors lead to poor water supply in the prairies and therefore limited pasture and nutrient availability [8]. This reduced nutrient availability is especially concerning during pregnancy, resulting in a null body weight increase and around a 22% decrease in body condition score in twin-bearing ewes [9].

The increase of twin-bearing ewes in the herd is assumed to be a good strategy to improve the productivity of sheep systems, since the additional newborn has a lower energy cost than the maintenance energy of an additional sheep. However, undernutrition of twin-bearing sheep may cause intrauterine growth restriction (IUGR), and therefore low birth weight (LBW) neonates, which suffer from increased morbidity and mortality [9]. In this case, under adverse climatologic and/or nutritional conditions like in the Magellan Patagonia, twin newborn mortality could reach up to 40% [10], negatively impacting profitability and animal welfare.

We recently demonstrated that IUGR in twin sheep fetuses is associated with hypoxia and oxidative stress [9]. Maternal oral supplementation with vitamins C and E during the pregnancy period (days 30 to 140 of gestation) increases these vitamins in the cord blood of near-to-term fetuses, diminishing the oxidative stress and increasing fetal weight [11]. In the present study, we aimed to evaluate the effects of maternal oral herbal vitamin C and E supplementation on pregnancy outcomes, postnatal viability, and development of lambs, from grazing twin-bearing ewes kept under natural underfed conditions or supplemented with concentrate.

## 2. Materials and Methods

### 2.1. Ethics Statement

The present study was carried out at the Instituto de Investigaciones Agropecuarias (INIA) research farm (Magellan Region, Chilean Patagonia, latitude 52°36′; longitude 70°56′) under standard commercial sheep breeding conditions. The protocol was approved by the Bioethics Review Committee of the Faculty of Veterinary Sciences of the University of Chile (Protocol # 11-2016) and by the Bioethics Committee of the Instituto de Investigaciones Agropecuarias (INIA), as institutions where the work was performed. Additional approval by the Bioethics Advisory Committee of the Chilean National Commission for Scientific and Technological Research (CONICYT), as funder of the project, was also obtained.

### 2.2. Animals and Experimental Procedure

In total, 120 twin-bearing Corriedale ewes were selected from the research farm’s commercial flock, following assessment of pregnancy rank by ultrasound at day 30 after mating (~20% of the total length of ovine pregnancy, estimated at a mean of 148 days) in synchronized cycles. Ewes were 4–6 years old, with a mean body weight (BW) of 57.5 ± 0.54 kg and mean body condition score (BCS, scale 0–5) of 2.0 ± 0.3 [12]. Ewes were mated over four days using rams with chests marked with a solution containing food-grade oil and colored earth. The exact day of service of the ewes was identified by daily visual inspection of the colored rumps of the ewes mated on the same day. Only females served in the first three days of the mating period were used. Animals were maintained in a paddock with natural pasture (*Festuca gracillima-Chiliotrichium diffusum*; Crude Protein: 3.3%, Metabolizable Energy: 1.9 Mcal/kg, Total Digestible Nutrients: 45%), with a stocking rate of 0.9 ewes per hectare and a dry matter availability of 525 kg per hectare, representative of Patagonian prairie conditions.

Pregnant ewes were randomly divided in four equal groups (*n* = 30 each) and ear tagged with different colors, according to each treatment. Group treatments were: P, control ewes consuming mainly natural pasture; P + V, ewes consuming natural pasture plus vitamin supplementation; P + S, ewes consuming natural pasture plus concentrate supplementation; P + VS, ewes consuming natural pasture plus vitamins and concentrate supplementation.

Supplementation with herbal vitamins was performed by including them as a premix in commercial concentrate feedstuffs (CP: 17.0%; ME: 3.0 Mcal/kg) containing vitamins C 10 g Kg^−1^ and E 7 g Kg^−1^ (C-Power™ and Herbal-E™, respectively, Nuproxa, Switzerland). Thus, sheep in the vitamin-treated groups (groups P + V and P + VS) received, daily and individually, 50 g of this product containing 500 mg of vitamin C and 350 IU vitamin E (doses previously shown to increase maternal and fetal plasma vitamin concentrations and antioxidant capacity [11]). Ewes from the control group (P) were fed daily with 50 g of concentrate without vitamins, with the purpose of equating the nutritional offer of the P + V group. Groups with nutritional supplementation (P + S and P + VS) initially received 450 g of the same concentrate formulation, but without additional vitamins. Supplementation began at 35.1 ± 0.1 days of gestation and continued until the first parturition occurred, to avoid stressing newborn lambs and their mothers with the supplementation process. The amount of concentrate offered was adjusted monthly after the ewes were weighed and BCS evaluated [13]. Concentrate intake was assessed daily in all groups and no refusals were observed for any of the treatment groups during any of the days the animals received supplementation. The lambing period was 7 days, with 82.6% of deliveries within 4 days. Following parturition, all animals received only a natural prairie diet. During the last week of pregnancy, the paddock where the ewes were kept was inspected four times a day to verify the presence of any recent parturition. When this happened, the newborn lambs were weighed, sexed, and ear tagged, then returned immediately to their mother and moved altogether to an adjacent paddock with similar pasture conditions. A sample of jugular blood (3 mL) was taken immediately after birth in six newborn lambs from each group and after being centrifuged plasma was stored in liquid nitrogen to be assayed for total antioxidant capacity (TAC).

Afterwards, BW and lamb survival were evaluated at mid-lactation (60 days of life) and weaning (120 days of life). Lamb survival was assessed as the difference between the number of born lambs and lambs alive at each time point measured.

### 2.3. Assessment of Total Antioxidant Capacity (TAC)

Assessment of TAC in newborn lamb plasma was performed using a colorimetric Antioxidant Assay Kit (Cayman Chemical Company, Ann Arbor, MI, USA), previously used for ovine plasma [9,11,14] according to the instructions of the manufacturer.

### 2.4. Statistical Analysis

The experimental model was a 2 × 2 factorial design, where feeding regimes (grazing and grazing plus concentrate supplementation) and vitamins supplementation (with and without vitamins supplementation) were the fixed effects. Comparisons were done by analysis of variance, using the general linear model procedure of SAS (GLM; SAS Institute Inc., Cary, NC, USA), after normality testing of the data. Changes in maternal BW and body condition score were assessed through repeated measures analysis, considering vitamin supplementation (with or without) and nutritional regimes (natural pasture or natural pasture plus supplement) as independent effects. Newborn data were analyzed using a linear model including the fixed effects of vitamins, nutritional plane, sex, and their interactions. The effect of newborn sex and treatment by sex interactions was not significant (*p* > 0.05) and therefore removed from the model. Lamb survival rate at each sampling time point was analyzed using the Wald test (mixed generalized linear models, InfoStat^®^, Córdoba, Argentina) The results are expressed as mean ± SEM and significant differences were considered when *p* < 0.05.

## 3. Results

### 3.1. Effects of Vitamins and Nutrition on Maternal Weight and Body Condition

**Figure 1:**
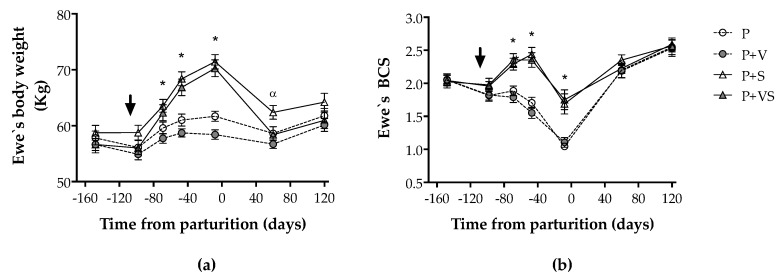
Mean (±SEM) body weight (**a**) and body condition score (BCS); (**b**) during pregnancy and subsequent lactation in twin-bearing ewes, maintained under extensive Magellan conditions and consuming only natural pasture (group P), natural pasture plus vitamin supplementation (group P + V), natural pasture plus concentrate supplementation (group P + S), and natural pasture plus vitamin and concentrate supplementation (group P + VS). The arrow shows the time when vitamins and/or nutritional supplementation begun. Asterisks indicate significant differences between sheep supplemented and non-supplemented with concentrate at the same sampling point, while symbol alpha indicates significant difference between P + S and P + V (*p* < 0.05).

Ewes without concentrate supplementation (groups P and P + V) showed a similar BW throughout the period of study. Ewes in both groups only showed a numerical, but not significant, increase around day 78 of pregnancy (day −70 from parturition) which remained until the end of the experimental period at day 120 after delivery (Figure 1a). Conversely, there was a significant increase in the BW of sheep in the groups supplemented with concentrate (groups P + S and P + VS) from day 78 of pregnancy (day −70 from parturition) until delivery (*p* < 0.05). The BW of these ewes decreased after parturition and reached similar values to non-supplemented sheep, with only P + S and P + V groups showing significant differences on day 60 after pregnancy (*p* < 0.05).

Body condition score (BCS) decreased through pregnancy in groups P and P + V whilst increasing at mid-pregnancy in groups P + S and P + VS. Hence, there were significant differences between supplemented and non-supplemented groups at days 78 and 100 of pregnancy (days –70 and –48 from parturition), but also near term (day 8 from parturition) despite a significant decrease in supplemented groups at this time (*p* < 0.05; Figure 1b). Afterwards, sheep gained BCS with no differences among groups at 60 and 120 days after delivery.

### 3.2. Effects of Maternal Nutrition and Vitamins on Birth-Weight and Antioxidant Status of Newborns

A total of 240 lambs were assessed, with a sex distribution of 51.6% females and 48.4% males. The effect of vitamins and/or nutritional supplementation, as well as their interaction, on the birth weight and antioxidant status of neonates are shown in Table 1. In brief, the mean birth weight for all groups was 4.04 ± 0.7 Kg, with no significant differences between females and males (4.04 ± 0.7 and 4.15 ± 0.7 Kg, respectively; *p* = 0.372). Birth weight was significantly increased by both vitamins (*p* < 0.005) and nutritional supplementation (*p* < 0.001), without interaction between them (*p* > 0.05).

Maternal vitamins supplementation also increased total antioxidant capacity of the newborns (*p* < 0.05) but there were no effects from the nutritional supplementation or interactions between vitamins and nutritional supplementations (*p* > 0.05).

### 3.3. Effects of Maternal Nutrition and Vitamins on Postnatal Viability and Development

There were no statistical differences in survival rate among lambs born to mothers with or without vitamin supplementation, nutritional concentrate, or both during pregnancy. Regardless, it is interesting to highlight that only the vitamin supplemented group (P + V) had survival rates about 17% and 14% higher than the counterparts without supplementation (P) at both 60 and 120 days of life, respectively. Similar results were obtained in the group whose mothers received nutritional and vitamin supplementation (Table 2).

The mean BW of the lambs from all groups was 22.7 ± 2.9 kg at 60 days old and 38.2 ± 4.2 kg at 120 days old, with no significant difference between females and males at 60 days (22.7 ± 3.14 kg vs. 22.7 ± 2.66, respectively; *p* = 0.655) or 120 days (37.6 ± 4.0 vs. 38.7 ± 4.4, respectively; *p* = 0.202). There were no significant effects of vitamin or nutritional supplementation on body weight at 60 or 120 days (Table 3), except a trend for vitamin supplementation at 120 days (*p* = 0.056).

## 4. Discussion

Pregnancy is associated with a significant increase in maternal BW caused by increases in fat deposition during early pregnancy, employed afterwards to support fetal development, and by the increase of fetal weight during late pregnancy [15]. This pattern is related to an increase in BCS due to fat accumulation during early pregnancy and a later decrease during late pregnancy. In the present experiment, such patterns were clearly observed in sheep supplemented with concentrate but not in non-supplemented ewes, in which BW showed non-significant changes and BCS was always decreasing throughout pregnancy. This finding supports evidence that twin-bearing ewes kept in a natural Magellan Patagonian prairie are normally raised in a state of undernutrition [9].

Supplementation with concentrate was adequate to keep maternal BCS above 2.0, a level still considered adequate for optimum lamb birth weight [16]. To this, it is important to highlight that the increase in BW observed in newborn lambs from mothers with nutritional supplementation is equivalent to the decrease in newborn lamb BW reported when the mothers consume a diet that only covers 70% of the requirements [17]. This coincides with the level of nutritional restriction suffered by pregnant sheep maintained under extensive grazing conditions in Magellan Patagonia, observed in the present study [18]. Accordingly, lamb birth weight from ewes supplemented only with concentrate increased around 17% when compared to lambs from non-supplemented mothers. This difference is consistent with what was previously reported as the final effect of maternal nutritional supplementation on fetal growth in underfed twin bearing ewes [9].

The results of the current study also confirmed that in underfed twin-bearing ewes kept on a natural prairie during pregnancy, the observed fetal growth restriction is associated with oxidative stress. We have previously shown that maternal undernutrition and twinning led to decreased oxygen supply and increased oxidative stress at the feto-placental unit in near-term pregnancies, which was associated with decreased intrauterine growth [9]. In this context, it has been demonstrated that maternal administration of antioxidant vitamins C and E results in an adequate placental transfer of both vitamins and improves fetal antioxidant status and growth in twin pregnancies [11]. The data from the present study are in agreement with the previous one, since supplementation with antioxidant herbal vitamins C and E improved birth weight and antioxidant capacity (in around 15% for both) of the lambs. There are, to the best of our knowledge, no other previous works on supplementation with vitamins C and E during gestation in ewes. There are, conversely, previous studies in sheep with vitamin E supplementation alone or combined with selenium during the last third of gestation. However, there are no conclusive results from these studies, since some show a positive effect on the lamb’s birth weight [19], while others failed to find any effect [20,21]. Such differences may be due to use of different doses of vitamin E, routes of administration, and duration of treatments, in addition to eventual differences in sheep pregnancy rank, breed, or other unspecified.

The main finding of the present study is that lambs born to ewes suffering significant losses of BCS during pregnancy, but supplemented with antioxidant vitamins, showed a birth weight similar to lambs from ewes with adequate BCS. Such data support results from an earlier study [9], indicating that the effect of carrying twins, plus its concomitant oxidative stress at the fetal-placental level, is even more limiting for intrauterine development than that of maternal undernutrition in pregnancies developed under the same disadvantageous conditions of Magellan Patagonia. We could infer, from these results, that the antioxidant vitamin supplementation is effective for counteracting placental dysfunctions affecting the nutrition and oxygenation of twin fetuses as a result of the fetal-placental oxidative stress.

These hypotheses are supported by previous data. Firstly, it has been found that the administration of antioxidants directly to sheep fetuses increased umbilical flow, which would favor fetal growth [22]. Supplementation with vitamin E to pregnant ewes has also been reported to increase angiogenesis, and therefore, would favor placental function and fetal development [23]. Such positive effects of antioxidant supplementation on placental efficiency and newborn body weight was reported in pregnant undernourished rats [24]. Similarly, in single ovine pregnancies developed under oxidative stress in a chronically hypoxic environment at high plateaus, supplementation with antioxidant vitamins increased placental efficiency between 10% and 45%, depending on the animal’s altitudinal origin [25].

On the other hand, it is widely accepted that postnatal growth is highly dependent on, and positively correlated with, birth weight [26,27]. However, we cannot leave aside some influence of compensatory growth; in this way, a former study in sheep showed that, under good nutritional conditions during the postnatal period, LBW lambs equaled the BW of normal birth weight lambs around 50 days of age [28]. In the present study, there were no differences in BW at 60 days of life among lambs from sheep with and without nutritional or vitamin supplementation.

Notwithstanding, lambs from mothers supplemented with vitamins were heavier (about 5%) at 120 days old than lambs from sheep with no treatment or even with concentrate supplementation. This finding is remarkable from the biological point of view, although this difference should be taken with caution because it was only at the limit of being statistically significant (*p* = 0.056). Previous studies on the effects of maternal supplementation with vitamin E in the last third of gestation on developmental patterns show contradictory results, reporting improvements in weaning weight [21], and no effects in other cases [20,29]. These results highlight the need for further studies on causes and significance of these effects, which are not specific of sheep. In this sense, studies in a different species, the pig, have shown that maternal supplementation with antioxidant compounds (polyphenols in this case), improves antioxidant status of the fetuses [30] and body weight of the neonates [31]. Afterwards, developmental patterns were positively affected by maternal supplementation independently of birth weight since, comparing individuals with similar weight at birth and weaning, piglets from supplemented sows reached higher average daily weight gain and, therefore, higher body weight and corpulence than their control counterparts [32].

Lamb viability was around 10%–12% higher in lambs from vitamin supplemented ewes, both at 60 and 120 days. These results, although differences did not reach statistical significance, have practical importance and support previous data addressing that lambs with higher birth weight have a lower risk of mortality and therefore greater viability in the pre-weaning stage [33]. These data are also supported by previous studies which showed that pre-weaning survival of twin lambs is around 10% increased by applying weekly injections of 900 IU of vitamin E during pregnancy [21] and around 70% increased by supplementation with vitamin E and selenium in the last third of gestation [20]. Hence, maternal supplementation with vitamins C and E in combination can also be a good strategy to improve the pre-weaning survival of lambs in multiple pregnancies. Effects from nutritional supplementation alone were lower than that of vitamins; undoubtedly the combination of both nutritional and vitamin supplementation leads to better postnatal survival.

Finally, it is important to note that the cost of vitamin supplementation per pregnant ewe was marginal in relation to the final benefit. The vitamin administration route used in this study, as a premix included in a commercial concentrate, meant a daily cost per animal of US $0.0195 including vitamins, resulting in a total cost for 113 days of gestation of US $2.20. However, the greatest cost in this form of supplementation is the concentrate, since vitamins represent less than 10% of the total cost. Thus, it is possible to include vitamins in lower amounts of the concentrate, which could significantly decrease the total value of the supplementation. Furthermore, it would be interesting to study eventual time windows during pregnancy in which supplementation for shorter periods could have similar effects to those found in the present study, certainly reducing its cost.

In conclusion, these results highlight the importance of preventing oxidative stress in compromised twin pregnancies from underfed ewes to improve newborn outcomes and later postnatal growth and viability of the lambs. Increases in prolificacy and productivity of the herd under natural rangeland systems by increasing twin-bearing ewes are penalized by a low birth weight and viability of the twins due to maternal undernutrition and increased feto-placental oxidative stress. In addition to nutritional supplementation of the pregnant sheep, administration of herbal antioxidant vitamins C and E during pregnancy may constitute a good nutritional strategy for sheep reared at harsh environmental conditions.

## Figures and Tables

**Table 1:** Effects of maternal nutritional plane and supplementation with vitamins C and E on newborn birth weight and antioxidant status in ewes kept in extensive grazing under Patagonian conditions.

Newborn Lamb Trait						*p*-Value	
	P	P + V	P + S	P + VS	V	NP	V × NP
Birth weight (Kg)	3.5 ± 0.2	4.0 ± 0.1	4.1 ± 0.1	4.3 ± 0.1	0.004	<0.001	ns
Plasma TAC at birth (mM Trolox equiv.)	0.37 ± 0.03	0.49 ± 0.02	0.43 ± 0.03	0.49 ± 0.02	0.030	ns	ns

Data are the mean ± SEM. Groups: P: control twin-bearing ewes consuming mainly natural pasture; P + V: twin-bearing ewes consuming natural pasture plus vitamin supplementation; P + S: twin-bearing ewes consuming natural pasture plus concentrate supplementation; P + VS: twin-bearing ewes consuming natural pasture plus vitamin and concentrate supplementation. V: vitamin effect; NP: nutritional plane effect; TAC: total antioxidant capacity; ns: not significant.

**Table 2:** Effects of maternal nutritional plane and vitamin C and E supplementation on survival rates at mid-lactation (60 days) and at weaning (120 days) of lambs born from twin-bearing ewes kept in extensive grazing under Patagonian conditions.

Lamb Survival Rates					*p*-Value
	P	P + V	P + S	P + VS	
Lambs survival at day 60 (%)	71.4	83.3	78.1	85.3	0.65
Lambs survival at day 120 (%)	70.0	79.9	65.9	79.4	0.79

P: control twin-bearing ewes consuming mainly natural pasture; P + V: twin-bearing ewes consuming natural pasture plus vitamin supplementation; P + S: twin-bearing ewes consuming natural pasture plus concentrate supplementation; P + VS: twin-bearing ewes consuming natural pasture plus vitamin and concentrate supplementation.

**Table 3:** Effects of maternal nutritional plane and vitamin C and E supplementation on body weight at mid-lactation (60 days) and at weaning (120 days) of lambs born from ewes kept in extensive grazing under Patagonian conditions.

						*p*-Value	
	P	P + V	P + S	P + VS	V	NP	V × NP
Body weight at day 60 (kg)	22.3 ± 0.6	22.6 ± 0.6	22.4 ± 0.6	23.5 ± 0.6	ns	ns	ns
Body weight at day 120 (kg)	37.3 ± 0.9	39.3 ± 1.4	37.4 ± 1.2	39.1 ± 0.6	0.056	ns	ns

Data are mean ± SEM. P: control twin-bearing ewes consuming mainly natural pasture; P + V: twin-bearing ewes consuming natural pasture plus vitamin supplementation; P + S: twin-bearing ewes consuming natural pasture plus concentrate supplementation; P + VS: twin-bearing ewes consuming natural pasture plus vitamin and concentrate supplementation. V: vitamin effect; NP: nutritional plane effect; ns: not significant.
